# Workforce Attrition Among Male and Female Physicians Working in US Academic Hospitals, 2014-2019

**DOI:** 10.1001/jamanetworkopen.2023.23872

**Published:** 2023-07-17

**Authors:** Ya-Wen Chen, Claudia Orlas, Tommy Kim, David C. Chang, Cassandra M. Kelleher

**Affiliations:** 1 Department of Surgery, Massachusetts General Hospital/Harvard Medical School, Boston, Massachusetts; 2 Codman Center for Clinical Effectiveness in Surgery, Massachusetts General Hospital/Harvard Medical School, Boston, Massachusetts; 3 Pediatric Surgery Trials and Outcomes Research Center, MassGeneral Hospital for Children, Boston, Massachusetts; 4 UMass Chan Medical School, Worcester, Massachusetts

## Abstract

**Importance:**

Retaining female physicians in the academic health care workforce is necessary to serve the needs of sociodemographically diverse patient populations.

**Objective:**

To investigate differences in rates of leaving academia between male and female physicians.

**Design, Setting, and Participants:**

This cohort study used Care Compare data from the Centers for Medicare & Medicaid Services for all physicians who billed Medicare from teaching hospitals from March 2014 to December 2019, excluding physicians who retired during the study period. Data were analyzed from November 11, 2021, to May 24, 2022.

**Exposure:**

Physician gender.

**Main Outcome and Measures:**

The primary outcome was leaving academia, which was defined as not billing Medicare from a teaching hospital for more than 1 year. Multivariable logistic regression was conducted adjusting for physician characteristics and region of the country.

**Results:**

There were 294 963 physicians analyzed (69.5% male). The overall attrition rate from academia was 34.2% after 5 years (38.3% for female physicians and 32.4% for male physicians). Female physicians had higher attrition rates than their male counterparts across every career stage (time since medical school graduation: <15 years, 40.5% vs 34.8%; 15-29 years, 36.4% vs 30.3%; ≥30 years, 38.5% vs 33.3%). On adjusted analysis, female physicians were more likely to leave academia than were their male counterparts (odds ratio, 1.25; 95% CI, 1.23-1.28).

**Conclusions and Relevance:**

In this cohort study, female physicians were more likely to leave academia than were male physicians at all career stages. The findings suggest that diversity, equity, and inclusion efforts should address attrition issues in addition to recruiting more female physicians into academic medicine.

## Key Points

**Question:**

Were there differences in rates of female and male physicians leaving academic medicine in the US from 2014 to 2019?

**Findings:**

In this cohort study of 294 963 physicians, female physicians were more likely to leave academic medicine compared with male physicians on adjusted analysis.

**Meaning:**

The findings suggest that diversity, equity, and inclusion efforts should address attrition in academic medicine.

## Teaser

This cohort study assesses difference in rates of female and male physicians leaving academic medicine in the US from 2014 to 2019.

## Introduction

The practice of academic medicine comes with unique responsibilities. Physicians practicing in academic hospitals not only treat patients but also are expected to conduct research for future patients. In addition, they play a key role in training future physicians. Attrition from academic practice, therefore, may endanger the quality of medicine in the future.

Attrition rates among academic physicians and among female physicians specifically are underresearched. The proportion of female physicians in academic medicine has been increasing, but the gender gaps in career opportunities and pay are still substantial.^1^ Women are less likely to hold senior faculty ranks or attain leadership positions than men and earn an estimated $2 million less than their male colleagues over a 40-year career.^1,2^ It is unclear whether the lack of parity in advancement in academic medicine will increase attrition rates or whether female physicians may prefer to stay in academia due to flexible schedules that accommodate other career goals, such as teaching or research, despite the obstacles in academic medicine.

Female physicians leaving academic practice may have important public health implications. Women bring unique perspectives to the practice of medicine, and their involvement in research and teaching can influence the future direction of the field.^3,4,5,6^ Therefore, the purpose of the study was to investigate gender differences in attrition rates from academia between male and female physicians practicing in academic hospitals across the US.

## Methods

### Data Characteristics

The cohort study was approved by the Mass General Brigham institutional review board and followed the Strengthening the Reporting of Observational Studies in Epidemiology (STROBE) reporting guideline.^7^ Informed consent was waived by the Mass General Brigham institutional review board because the research presented no more than minimal risk of harm to participants and involved no procedure for which written consent is normally required outside the research context. This study was performed using Care Compare data publicly downloaded from the Centers for Medicare & Medicaid Services in October 2021.^8^ This file contains general information about physicians and clinicians who bill Medicare, including their demographic information, medical school attended, graduation year, clinical specialty, and institutional affiliation.

### Inclusion and Exclusion Criteria

All physicians who billed Medicare from a teaching hospital in March 2014 were included in this study and were followed up until December 2019. The year 2014 was selected because it had the earliest available data at the time of the study; data from 2020 and 2021, although available, were not included to avoid confusion with COVID-19–related disruption to clinical practices.

We used the teaching hospital definition adopted by Medicare to define an academic hospital, noted as a hospital that received payment for Medicare direct graduate medical education, inpatient prospective payment system indirect medical education, or psychiatric hospital indirect medical education programs.^9^ We chose this definition because of its objectivity and recognize that there could be other definitions for academic hospitals and that not all teaching hospitals have residencies in every clinical specialty. We also acknowledge that not every physician in such a teaching hospital has a teaching and research responsibility in addition to their clinical practice.

Physicians who retired during the study period were excluded. A physician was considered to be retired if their last year of Medicare billing from any institution was more than 35 years after their medical school graduation year, which would be an average age of retirement of 65 years.^10^

### Primary Variables

The primary outcome was leaving academia and was defined as not billing Medicare from a teaching hospital for more than 1 year. The primary independent variable was physician gender. Physician gender was self-reported and was collected from the database directly.

### Statistical Analysis

Data were analyzed from November 11, 2021, to May 24, 2022. Unadjusted comparison for categorical variables was performed using a χ^2^ test and for continuous variables using a *t* test. Multivariable logistic regression was conducted, adjusting for physician experience defined as years since medical school graduation, graduating medical school ranking (top 20 vs not top 20, according to 2022 *US News and World Report* research medical school list),^11^ specialty (surgical vs nonsurgical), and region of the country (Northeast, Midwest, South, and West).^12^ Surgical specialties included surgical oncology, thoracic surgery, vascular surgery, urology, orthopedic surgery, neurosurgery, maxillofacial surgery, hand surgery, general surgery, colorectal surgery, and cardiac surgery. Stratified subset analyses were further performed to compare gender differences in attrition rate from academia among physicians for the aforementioned variables.

A geospatial analysis was also performed. The odds ratios (ORs) of leaving academia comparing female with male physicians across states are depicted on a US map (eFigure in Supplement 1). All analyses were performed in Stata, version 15.1 (StataCorp LLC) and ArcGIS Pro, version 2.9. Statistical significance was defined as 2-sided *P* < .05 for primary analysis and 2-sided *P* < .01 for secondary analyses to avoid type I errors.

## Results

A total of 294 963 physicians were included in this study, of whom 69.5% were men and 30.5% were women. Among all physicians, 30.4% were within 15 years of medical school graduation, 44.3% were 15 to 29 years from graduation, and 25.3% were 30 years or more from graduation. A total of 12.2% of the physicians were surgeons, and 11.3% of the physicians graduated from a top-20 medical school. The overall attrition rate from academia during the study period for the entire study cohort was 34.2% (Table 1).

**Table 1.** Characteristics of Physicians^a^

Characteristic	Physicians, No (%)			*P* value
	All (N = 294 963)	Female (n = 89 965)	Male (n = 204 998)	
Left academia^b^	100 937 (34.2)	34 494 (38.3)	66 443 (32.4)	<.001
Time since medical school graduation, y				
<15	89 778 (30.4)	37 252 (41.4)	52 526 (25.6)	<.001
15-29	130 648 (44.3)	41 017 (45.6)	89 631 (43.7)	
≥30	74 537 (25.3)	11 696 (13.0)	62 841 (30.7)	
Graduated from top-20 medical school	33 359 (11.3)	10 199 (11.3)	23 160 (11.3)	.76
Surgical specialty	36 063 (12.2)	4220 (4.7)	31 843 (15.5)	<.001
Region				
Northeast	78 758 (26.7)	25 625 (28.5)	53 133 (25.9)	<.001
Midwest	73 462 (24.9)	22 823 (25.4)	50 639 (24.7)	
South	89 009 (30.2)	24 765 (27.5)	64 244 (31.3)	
West	52 805 (17.9)	16 520 (18.4)	36 285 (17.7)	

^a^ Physicians who retired during the study period were excluded. Physician retirement was considered when a physician’s last year of Medicare billing was more than 35 years after their medical school graduation year.

^b^ Leaving academia was defined as not having billed Medicare from a teaching hospital for more than 1 year.

Compared with male physicians, female physicians were more likely to be within 15 years from medical school graduation and less likely to be surgeons. The attrition rate among women during the study period was significantly higher than that among men (38.3% vs 32.4%; *P* < .001) (Table 1). Attrition rates among women were significantly higher than those among male physicians at every stage of their careers (time since medical school graduation: <15 years, 40.5% vs 34.8% [*P* < .001]; 15-29 years, 36.4% vs 30.3% [*P* < .001]; ≥30 years, 38.5% vs 33.3% [*P* < .001]). Of note, both female and male surgeons were less likely to leave academia in the midcareer stage than during other career stages.

The ORs of attrition rates from academia comparing female with male physicians by state are given in Table 2 and the eFigure in Supplement 1. Female physicians were more likely to leave academia than were male physicians in 37 states and in Washington, DC, and Puerto Rico; there was no gender difference in odds of leaving academia in 13 states. The ORs of leaving academia were between 0.95 and 1.05 in 3 states (Alaska, Nebraska, and South Dakota), and there was no difference in odds of leaving academia between female and male physicians in these states. The ORs were greater than 1.05 in all other states; Washington, DC; and Puerto Rico. There were no states with an OR less than 0.95.

**Table 2.** Adjusted Odds Ratios of Female Physician vs Male Physician Leaving Academia by State

State	Adjusted odds ratio (99% CI)
Alaska	0.96 (0.58-1.61)
Alabama	1.29 (1.06-1.58)
Arkansas	1.75 (1.39-2.21)
Arizona	1.28 (1.09-1.50)
California	1.35 (1.27-1.44)
Colorado	1.38 (1.19-1.60)
Connecticut	1.45 (1.26-1.66)
Delaware	1.57 (1.08-2.30)
Florida	1.38 (1.27-1.51)
Georgia	1.45 (1.28-1.64)
Hawaii	1.55 (1.18-2.04)
Iowa	1.22 (1.00-1.49)
Idaho	1.09 (0.80-1.49)
Illinois	1.27 (1.16-1.39)
Indiana	1.32 (1.14-1.53)
Kansas	1.33 (1.06-1.68)
Kentucky	1.33 (1.11-1.59)
Louisiana	1.35 (1.13-1.60)
Massachusetts	1.18 (1.08-1.29)
Maryland	1.46 (1.28, 1.66)
Maine	1.28 (0.98-1.66)
Michigan	1.27 (1.16-1.39)
Minnesota	1.33 (1.18-1.51)
Missouri	1.22 (1.05-1.40)
Mississippi	1.42 (1.07-1.90)
Montana	1.40 (0.96-2.04)
North Carolina	1.37 (1.22-1.53)
North Dakota	1.35 (0.95-1.90)
Nebraska	1.03 (0.80-1.32)
New Hampshire	1.13 (0.84-1.53)
New Jersey	1.34 (1.21-1.48)
New Mexico	1.26 (0.99-1.62)
Nevada	1.37 (1.05-1.78)
New York	1.30 (1.22-1.38)
Ohio	1.22 (1.12-1.34)
Oklahoma	1.32 (1.08-1.63)
Oregon	1.26 (1.06-1.49)
Pennsylvania	1.38 (1.27-1.49)
Puerto Rico	2.69 (1.89-3.83)
Rhode Island	1.15 (0.90-1.49)
South Carolina	1.36 (1.14-1.62)
South Dakota	1.00 (0.68-1.46)
Tennessee	1.31 (1.12-1.52)
Texas	1.23 (1.14-1.33)
Utah	1.18 (0.92-1.51)
Virginia	1.39 (1.22-1.57)
Vermont	1.32 (0.89-1.94)
Washington	1.20 (1.07-1.35)
Washington, DC	1.52 (1.19-1.93)
Wisconsin	1.24 (1.09-1.40)
West Virginia	1.55 (1.20-2.00)
Wyoming	1.34 (0.76-2.38)

The adjusted analysis is presented in Table 3. Female physicians were more likely to leave academia than were male physicians (OR, 1.25; 95% CI, 1.23-1.28). In addition, physicians in their middle career, surgeons, and physicians who graduated from a top-20 medical school had a lower likelihood of leaving academia. Notably, physicians in the South and West regions of the country were more likely to leave academia.

**Table 3.** Adjusted Odds Ratios for Physicians Leaving Academia^a^

Characteristic	Adjusted odds ratio (95% CI)	*P* value
Gender		
Male	1 [Reference]	NA
Female	1.25 (1.23-1.28)	<.001
Time since medical school graduation, y		
<15	1 [Reference]	NA
15-29	0.84 (0.82-0.85)	<.001
≥30	0.96 (0.94-0.98)	<.001
Graduated from top-20 medical school		
No	1 [Reference]	NA
Yes	0.85 (0.83-0.87)	<.001
Primary specialty		
Nonsurgical	1 [Reference]	NA
Surgical	0.68 (0.66-0.70)	<.001
Region		
Northeast	1 [Reference]	NA
Midwest	1.00 (0.98-1.03)	.72
South	1.28 (1.26-1.31)	<.001
West	1.69 (1.65-1.73)	<.001

Abbreviation: NA, not applicable.

^a^ Leaving academia was defined as not having billed Medicare from a teaching hospital for more than 1 year.

The subset analysis was further stratified by physician’s graduating medical school, experience, specialty, and geographic area (Figure). Female gender was independently associated with leaving academia at every career stage, in both surgical and medical specialties, in all regions of the country, and regardless of the graduating medical school.

**Figure.**
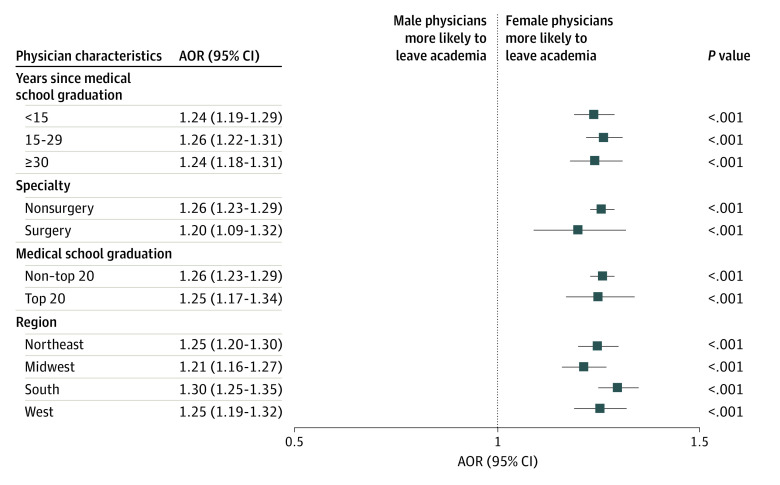
Subgroup Analyses of Gender Differences in Attrition Rates Markers represent adjusted odds ratios (AORs), and horizontal lines represent 95% CIs.

## Discussion

Our study found that female physicians were more likely to leave academia at all career stages, in all regions of the country, in both surgical and nonsurgical specialties, and regardless of graduating medical school ranking in the US between 2014 and 2019. With the physician shortage projected to reach 124 000 physicians by 2034 and the cumulated resources required to train and recruit physicians in academia, interventions to retain physicians, especially female physicians, are crucially needed.^3,4,5^

Our study focused on the gender differences in attrition rates from academia among practicing physicians in academic hospitals. Similar to our finding, a prior Association of American Medical Colleges report on attrition of medical school faculty showed a higher attrition rate among female faculty than among male faculty (10-year attrition rate: 44% vs 42%), with an overall rate estimated to be 43% in the 2010 cohort.^13^ With a shorter follow-up period and a focus on practicing physicians, our study demonstrated 38.3% and 32.4% 5.5-year attrition rates from academia for female and male physicians, respectively, in the 2014 cohort. These findings highlight the importance of developing targeted strategies for retaining female physicians in academia to ensure diverse representation and viewpoints.^14^

Our study adds to the literature by addressing the hypothesis that has been proposed to explain why women may have higher attrition rates from academia. It is often suggested that family obligations are the main factor associated with female physicians leaving academic hospitals.^15,16,17^ It has been argued that since women are the main caregiver of the family, a disproportionate work-family conflict may lead women to leave academic hospitals more often than men.^18,19^ However, we found that higher attrition rates from academia among women persisted throughout all career stages, suggesting that family obligations were unlikely to explain this gender disparity. If family obligation was the main factor associated with more women leaving academic hospitals, we would expect to see the difference decrease among the late-career physicians when female physicians have fewer domestic duties. This, however, was not found in our study. Therefore, our findings suggest that there may be other factors associated with the higher attrition rate from academia among female physicians.

One of these factors may be the more difficult work environment encountered by female physicians in academic hospitals. The literature has highlighted many challenges, ranging from microaggression to frank discrimination and harassment from peers, staff, and even patients toward female physicians.^20,21^ Female physicians also lack the same career-building opportunities, sponsorships, and role models as their male counterparts.^1,22,23,24,25^ For instance, female surgeons receive fewer new patient referrals and perform less complex operations than their male peers despite having equivalent availability, training, and seniority.^26,27,28^ Inequities in the work environments between men and women have also been documented. Female physicians are promoted at a slower rate, are less likely to be in leadership positions, and receive less compensation than male physicians.^24^ Uneven distribution of academic and research support, such as time and space, funding, or personnel assistance, further contributes to the problem.^29,30^ Furthermore, female physicians are more likely to be asked to volunteer for tasks and committees that are not relevant to or not beneficial for career advancement than their male colleagues.^31^ These tasks may preclude female physicians from conducting complex administrative or research projects and may further deepen the gender gap in promotion and leadership opportunities. This leads to a lack of female leaders to champion systemic changes, which in turn maintains the status quo and perpetuates inequities. Further investigations into how these inequities may specifically contribute to higher female attrition rates are needed.

Of interest, we found that attrition rates from academia were lower among mid-career physicians. Although mentorship and career-building programs are often targeted to support early-career physicians, our data suggest that there are still opportunities for improvement. Additionally, succession and transition plans for late-career physicians may help retain early-career physicians in academia. We also found lower attrition rates from academia among graduates from medical schools identified to be top 20. This finding may be expected given the emphasis on the research mission among these medical schools. Surgeons had lower attrition rates from academia than internists, which may suggest that internists have more flexibility in finding opportunities outside academic hospitals. Our study found a significant geographic difference in overall attrition rates from academia and in the gender gap of the attrition rates. Physicians in the West were more likely to leave academia in general, and the gender gap in the South was greater than in other regions. These trends suggest that some external modifiable factors, such as the inclusiveness of the work environment and opportunities for nonacademic careers, may be associated with attrition. Future studies should investigate social and health system characteristics that might affect attrition from academia in general and among women specifically within and outside academic hospitals.

This study has important implications for the future workforce in medicine. Despite equal numbers of women and men graduating from medical school, the higher attrition rate from academia among women may lead to decreasing representation of women in the academic workforce. Therefore, an action plan to retain female academic physicians, the teachers and researchers meant to care for patients now and in the future, is essential to maintaining a diverse and representative academic workforce.

### Strengths and Limitations

Our study has strengths. First, this was a nationwide study with a large sample size, while most prior studies were from single institutions. Thus, it provides evidence for the national context. Second, our study was based on objective data, while most prior studies involved surveys investigating leaving or the intent to leave academic medicine.^15,32^ Our study, therefore, was less likely to be influenced by respondent or selection bias.

Our study also has some limitations. First, our study only included physicians who billed Medicare. Therefore, physicians who did not see patients in the Medicare-eligible age ranges, such as pediatricians, or physicians who did not accept Medicare were not captured in our study. However, it has been shown that 93% of nonpediatric primary care physicians accept Medicare, suggesting that our study likely captured the majority of physicians nationally.^22^ Second, we did not have data on the percentage effort of each individual across different academic obligations, and their academic track (research, clinical, or teaching) may have affected the likelihood of leaving. However, physicians practicing in academic hospitals commonly perform some education or research work, although with varying degrees of commitment.

## Conclusions

In this cohort study, female physicians were more likely to leave academic medicine than were male physicians in the US from 2014 to 2019. This gender gap existed in all career stages, in all regions of the country, in both surgical and nonsurgical specialties, and regardless of graduating medical school ranking. The findings suggest that research on whether inequities in the work environment of academic medicine are associated with the disparity in attrition rates is needed. Future efforts in retaining female physicians in academia should address system-level workplace inequities.

## Notes

Supplement 1. **eFigure.** Odds Ratios of Attrition Rates Comparing Female Vs Male Physicians Across States

Supplement 2.
Data Sharing Statement
