# Supplementary material for: Workforce Attrition Among Male and Female Physicians Working in US Academic Hospitals, 2014-2019
Source: JAMA Netw Open. 2023 Jul 17;6(7):e2323872. doi: 10.1001/jamanetworkopen.2023.23872 (PMC10352856; doi:10.1001/jamanetworkopen.2023.23872)
Supplement: Supplement 1. — eFigure. Odds Ratios of Attrition Rates Comparing Female Vs Male Physicians Across States [file jamanetwopen-e2323872-s001.pdf]

Supplemental Online Content

Chen YW, Orlas C, Kim T, Chang DC, Kelleher CM. Workforce attrition among male and female physicians working in US academic hospitals, 2014-2019. *JAMA Netw Open.* 2023;6(7):e2323872. doi:10.1001/jamanetworkopen.2023.23872

eFigure. Odds Ratios of Attrition Rates Comparing Female Versus Male Physicians Across States

This supplemental material has been provided by the authors to give readers additional information about their work.

© 2023 Chen YW et al. *JAMA Network Open.*

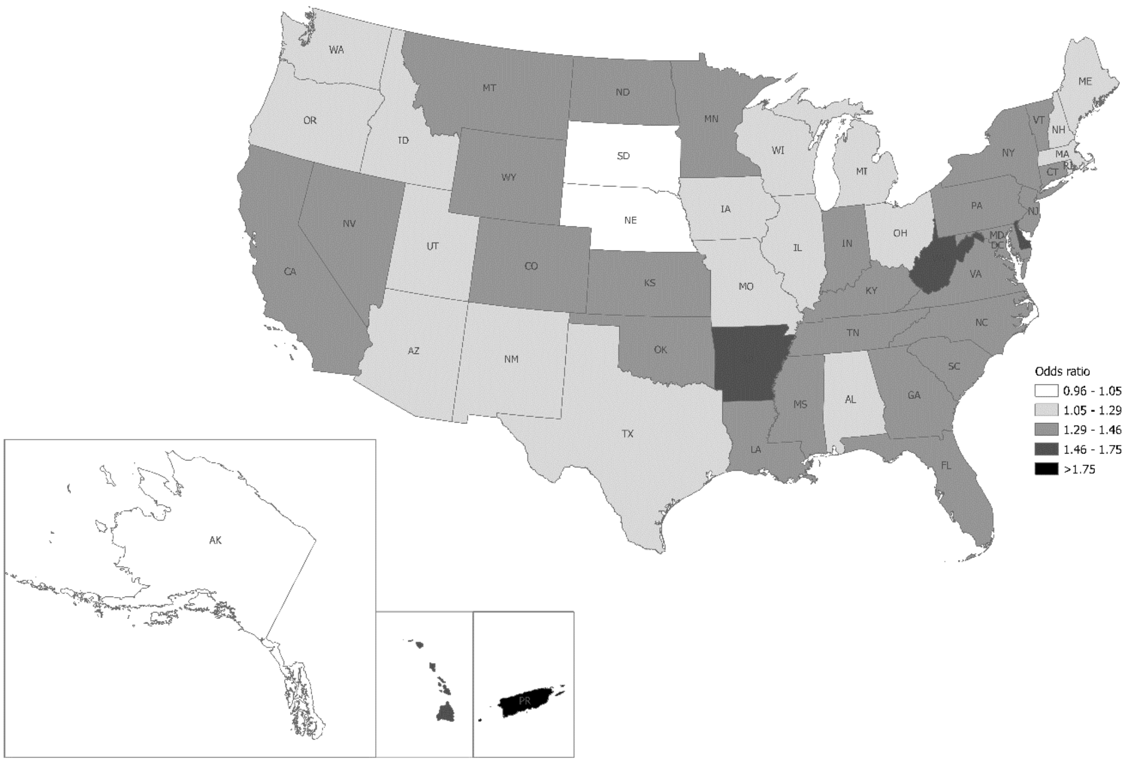

eFigure. Odds Ratios of Attrition Rates Comparing Female Versus Male Physicians Across States

© 2023 Chen YW et al. *JAMA Network Open.*
