# Supplementary material for: Workforce Attrition Among Male and Female Physicians Working in US Academic Hospitals, 2014-2019
Source: JAMA Netw Open. 2023 Jul 17;6(7):e2323872. doi: 10.1001/jamanetworkopen.2023.23872 (PMC10352856; doi:10.1001/jamanetworkopen.2023.23872)
Supplement: Supplement 2. — Data Sharing Statement [file jamanetwopen-e2323872-s002.pdf]

Data Sharing Statement

Chen. Workforce Attrition Among Male and Female Physicians Working in US Academic Hospitals, 2014-2019. *JAMA Netw Open*. Published July 17, 2023.
doi:10.1001/jamanetworkopen.2023.23872

Data
Data available: No
